# Small Molecule of Secreted Clusterin Promotes Mitochondrial Function, Synaptic Plasticity and Improved Memory in an Alzheimer's Disease Mouse Model

**DOI:** 10.1002/alz70859_104473

**Published:** 2025-12-25

**Authors:** Samar Padder, Whitaker Cohn, Jesus J Campagna, Dongwook Wi, Jessica Lee, Gazmend Elezi, Sahiba Beniwal, Chunni Zhu, Barbara Jagodzinska, Patricia Spilman, Julian Whitelegge, Varghese John

**Affiliations:** ^1^ University of California, Los Angeles (UCLA), Los Angeles, CA USA

## Abstract

**Background:**

Clusterin (CLU) is a key genetic risk factor for late‐onset Alzheimer’s disease (AD). Its secreted clusterin isoform (sCLU) is neuroprotective, promoting amyloid beta (Aβ) and tau clearance, reducing oxidative stress, and modulating immune responses, while the nuclear form (nCLU) is neurotoxic. Reduced sCLU levels, associated with specific CLU gene variants, exacerbate cognitive decline and protein aggregation. This study describes identification small molecules that enhance sCLU levels in vitro and in vivo in AD models, offering a novel therapeutic approach for AD.

**Method:**

A high‐throughput screening (HTS) campaign in U‐87 MG glioblastoma cells identified compounds increasing sCLU expression. Medicinal chemistry optimized hit candidates for potency as sCLU enhancers, brain permeability, and drug‐like properties. Pharmacokinetics, pharmacodynamics, and preclinical acute and chronic efficacy were evaluated in ApoE4TR‐5XFAD and 3xTg‐AD mouse models. The lead candidate, DDL‐357, was assessed for sCLU, Aβ, and p‐Tau modulation and memory improvement in Barnes maze tests. Global proteomics of hippocampal tissue explored molecular mechanisms. A neurite outgrowth assay in iPSC‐derived human neurons measured neuronal development effects.

**Result:**

Inhibition of histone deacetylases (HDACs) and bromodomain and extra‐terminal (BET) proteins were identified as mechanisms for sCLU enhancement. BET inhibitors showed greater potency than HDAC inhibitors, increasing sCLU at low nanomolar concentrations without notable toxicity. DDL‐357, a BET inhibitor, demonstrated strong brain penetration, stability, and significant sCLU elevation in ApoE4TR‐5XFAD mice after subchronic treatment. Chronic treatment of DDL‐357 in 3xTg‐AD mice reduced phosphorylated tau at Ser396 and improved spatial learning and memory in Barnes maze testing. Proteomic analysis revealed upregulation of proteins essential for mitochondrial health, synaptic plasticity, and protein homeostasis. DDL‐357 also increased mitochondrial oxygen consumption, sirtuin‐1 expression, and neurite outgrowth in human neurons.

**Conclusion:**

This study introduces a novel class of sCLU‐enhancing compounds, highlighting a promising strategy to mitigate AD pathology. DDL‐357 shows potential to address primary drivers of AD while supporting neuroprotective mechanisms and improving memory in AD models. These findings support further development of DDL‐357, with future studies potentially including larger preclinical cohorts, expansion to rat models, and additional biomarker analysis.

